# Mit Apps gegen den Tinnitus? Ein systematisches Review zu Qualität, Interventionselementen und Techniken der Verhaltensänderung

**DOI:** 10.1007/s00103-023-03805-1

**Published:** 2023-12-12

**Authors:** Alina Rinn, Sandy Hannibal, Sarah Goetsch, Cornelia Weise, Dirk Lehr

**Affiliations:** 1 Fachbereich Psychologie, AE Klinische Psychologie und Psychotherapie, Philipps-Universität Marburg, Gutenbergstraße 18, 35032 Marburg, Deutschland; 2 Abteilung für Gesundheitspsychologie und Angewandte Biologische Psychologie, Leuphana Universität Lüneburg, Lüneburg, Deutschland

**Keywords:** Tinnitus, Internetbasierte Interventionen, Smartphone-Apps, Interventionselemente, Behavior Change Techniques, Tinnitus, Internet-based interventions, Smartphone apps, Intervention components, Behavior change techniques

## Abstract

**Hintergrund:**

Über App-Stores werden Applikationen (Apps) für Tinnitusbetroffene angeboten und versprechen schnelle Hilfe gegen die Ohrgeräusche. Für Betroffene und Behandelnde ist es jedoch gleichermaßen schwierig, deren Qualität, Potenziale oder Risiken einzuschätzen. Ziel dieser Studie war es, eine Übersicht zu deutschsprachigen Tinnitus-Apps zu erstellen und damit eine Orientierung für Forschung und Versorgung zu geben.

**Methoden:**

Es wurde eine systematische Recherche (November 2020–April 2021) in den umsatzstärksten Stores (Google Play Store und Apple App Store) durchgeführt. Spezifisch für Tinnitus entwickelte Apps wurden von unabhängigen Wissenschaftlerinnen multiperspektivisch bewertet: (a) ein Qualitäts-Screening erfolgte anhand der deutschen Version der Mobile App Rating Scale (MARS-G), (b) Interventionselemente wurden mittels neu entwickelten Kategoriensystems und (c) implementierte Strategien für aktives Tinnitus-Management anhand von Techniken der Verhaltensänderung (Behavior Change Techniques, BCTs) analysiert.

**Ergebnisse:**

Die Suche ergab 1073 Apps, wobei 21 Apps in die Analyse eingingen. Die Apps wiesen nach MARS‑G eine mittlere Qualität auf (M = 3,37; SD = 0,39). Die meisten Apps boten Geräusche (*n* = 18) und Informationen (*n* = 9) an oder erfassten Charakteristika des Tinnitus (*n* = 13). 24 der 93 BCTs wurden mindestens einmal identifiziert. Nur eine App wurde in nicht-randomisierten Studien evaluiert.

**Diskussion:**

Wenngleich eine Vielzahl von Apps vorliegt, fokussiert die Mehrzahl auf wenige Elemente (z. B. Geräusche und Informationen). Das Potenzial, über die Apps wichtige, evidenzbasierte Inhalte zur Tinnitusbewältigung zu vermitteln, wird damit nicht ausgeschöpft. Die multiperspektivische Evaluation zeigt Verbesserungspotenzial für Tinnitus-Apps auf.

**Zusatzmaterial online:**

Zusätzliche Informationen sind in der Online-Version dieses Artikels (10.1007/s00103-023-03805-1) enthalten.

## Abstract (en)

**Background:**

Various smartphone applications (apps) for people with tinnitus are available and promise tinnitus relief. Yet, it is difficult for people suffering from tinnitus and healthcare professionals to estimate their quality, potential, or risks. The current study aims to generate an overview of available tinnitus apps in the German language and to offer orientation for research and healthcare providers.

**Method:**

The most prominent stores were searched systematically (November 2020–April 2021; Google Play Store and Apple App Store). Apps specifically developed for tinnitus were evaluated by two independent raters using multiple approaches: a) a quality screening was performed using the German version of the Mobile App Rating Scale (MARS-G), b) intervention components were assessed using a newly developed list of categories, and c) implemented strategies for active tinnitus management were analyzed using behavior change techniques (BCTs).

**Results:**

The search yielded 1073 apps, of which 21 apps were analyzed. The apps’ overall quality as assessed by MARS‑G was average (M = 3.37, SD = 039). A lot of apps offered sounds (*n* = 18) and information (*n* = 9) or assessed tinnitus characteristics (*n* = 13). Out of 93 BCTs, 24 were identified at least once. Only one app was evaluated in non-randomized trials.

**Conclusion:**

Although a variety of apps are available, most of them focus on a few intervention components (e.g., sounds or information). Therefore, apps do not exploit their potential to impart important evidence-based content for tinnitus management. The app evaluation using multiple approaches points out potential for improvement.

## Hintergrund

Tinnitus ist gekennzeichnet durch die Wahrnehmung eines Tons ohne korrespondierenden externalen Stimulus. In der Allgemeinbevölkerung sind 10–15 % von Ohrgeräuschen betroffen [1]. Ein Teil der Betroffenen ist durch den Tinnitus erheblich beeinträchtigt (bis zu 4 %; [2–4]). Die Ohrgeräusche können mit Konzentrationsproblemen, Schlafstörungen, Depression und Angststörungen einhergehen [5, 6]. Die weite Verbreitung von Smartphones und die vielfältigen technologischen Möglichkeiten, mobile Anwendungen (Applikationen, Apps) zu gestalten, stellt ein großes Potenzial dar, um den Zugang zu evidenzbasierten Interventionen zu erleichtern und damit langfristig die Versorgungssituation zu verbessern [7–9]. Online-Interventionen und Apps könnten als wirksames Selbsthilfeinstrument genutzt werden, aber auch eine sinnvolle Ergänzung von etablierten Therapieverfahren darstellen (z. B. Begleitung der ambulanten Behandlung, Überbrückung von Wartezeiten auf Psychotherapie). Damit haben sie das Potenzial, Kosten zu reduzieren und die Wirksamkeit von Behandlungen zu verbessern [10]. Gleichzeitig birgt der Einsatz von internetbasierten Verfahren neue Herausforderungen und potenzielle Risiken. So sind Datensicherheit, Qualität, Wirksamkeit, Risiken in der Anwendung bzw. mögliche negative Effekte wichtige Aspekte, die berücksichtigt werden müssen [10–12].

Die große Zahl von Tinnitus-Apps weist auf das Bedürfnis der Betroffenen nach niedrigschwelligen und leicht zugänglichen Unterstützungsangeboten hin [8, 13–16]. Für Betroffene und Behandelnde ist es jedoch gleichermaßen schwierig, die Qualität von Gesundheits-Apps einzuschätzen. Laut einer aktuellen Studie [17] erlauben die Beschreibungen in den App-Stores in 70–80 % der analysierten Apps keine Qualitätsbewertung anhand medizinischer Kriterien. Auch die User-Bewertungen sind keine verlässliche Quelle für Qualitätseinschätzungen [18]. Vor diesem Hintergrund wurden verschiedene Instrumente entwickelt, die eine erste orientierende Bewertung der Qualität von Gesundheits-Apps durch unabhängige Expert:innen erlauben [7, 19, 20]. Die häufig eingesetzte Mobile App Rating Scale (MARS) fungiert als Qualitätsscreeninginstrument [20, 21].

Während international bereits erste Übersichtsarbeiten zur Qualität von Apps auf Basis der MARS existieren, die auch Apps für Tinnitusbetroffene berücksichtigen [8, 16, 22], liegt für deutschsprachige Apps keine systematische Übersicht vor. In den 3 Studien ist die Varianz in der Qualität der Apps auffällig, wobei die Qualität der Funktionalität (z. B. Usability) höher ausfällt als beispielsweise die Qualität der Informationen. Einschränkend ist zu erwähnen, dass in einer der Studien nicht nur Apps für Tinnitus, sondern auch Apps zur Vermittlung von Strategien der kognitiven Verhaltenstherapie (KVT) analysiert wurden [16], dass in der zweiten Studie die Generalisierbarkeit der Ergebnisse durch eine Vorauswahl beliebter Apps einschränkt wird [8] und dass in die dritte Studie Apps für Tinnitus gemeinsam mit Apps gegen Gleichgewichtsstörungen in die Analyse eingingen [22].

Während Instrumente wie die MARS über allgemeine Qualitätsmerkmale informieren und dabei auf digitale Merkmale fokussieren, sind Informationen zu den konkreten, krankheitsspezifischen Interventionselementen einer App nicht abgedeckt. Insbesondere für Behandelnde ist dies jedoch wichtig, um beispielsweise Apps zur Selbsthilfe zu empfehlen oder sie in ein therapeutisches Gesamtkonzept integrieren zu können [8]. Bisherige Studien beschreiben die implementierten Interventionselemente in Apps mit vergleichsweise groben Kategorien, z. B. „tinnitus management“, „tinnitus assessment and measurement“ oder „sound therapy“ [13, 14, 16], was die Aussagemöglichkeit zu deren konkreten Inhalten einschränkt. Eine detaillierte Analyse der Interventionselemente in Tinnitus-Apps findet sich bei Sereda und Kolleg:innen [8]. Allerdings handelte es sich in dieser Studie um eine Auswahl beliebter Apps zum Tinnitusmanagement, die aber nicht unbedingt spezifisch für Tinnitus entwickelt wurden (z. B. auch Apps zur Förderung von Schlaf, Entspannung). Eine systematische und detaillierte Analyse der über Apps angebotenen Interventionselemente (z. B. Nutzung von Hörtaktiken, Einsatz von Geräuschen oder Übungen zur Aufmerksamkeitslenkung) wurde nach unserem Wissen bislang weder national noch international vorgenommen.

Eine dritte Perspektive auf Apps bietet die Analyse der eingesetzten Techniken der Verhaltensänderung (Behavior Change Techniques [BCTs]) von Michie und Kollegen [23]. Mit dieser Taxonomie wurde ein vielbeachteter Standard entwickelt, der diagnoseübergreifend die Identifikation der „active ingredients“ von komplexen Interventionen erlauben soll. Die Beschreibung von Interventionen entlang der eingesetzten BCTs verspricht (a) Replikationen in der Interventionsforschung zu erleichtern, (b) die originalgetreue Implementierung wirksamer Interventionen sicherzustellen, (c) systematische Reviews und Metaanalysen zur Wirksamkeit dieser Techniken zu ermöglichen, (d) die Entwicklung von Interventionen zu erleichtern und (e) die Wirkmechanismen besser zu verstehen, um Interventionen systematisch und kontinuierlich verbessern zu können [23]. Während das National Institute for Health Care and Excellence (NICE) im Vereinigten Königreich von Anbieter:innen digitaler Anwendungen explizit verlangt, dass evidenzbasierte BCTs bei der Interventionsentwicklung berücksichtigt und dokumentiert werden [24], gilt dies in Deutschland bislang nur für Anbieter:innen von digitalen Präventions- und Gesundheitsförderungsangeboten in der Individualprävention [25]. Eine Analyse der in Tinnitus-Apps eingesetzten BCTs liegt bislang nicht vor.

Ziel dieser Untersuchung war es daher: a) verfügbare Tinnitus-Apps in deutscher Sprache zu identifizieren, b) die Qualität anhand der deutschen Version der MARS‑G [21] zu analysieren sowie c) enthaltene Interventionselemente und d) eingesetzte BCTs [23] zu identifizieren.

## Methoden

Die Studie wurde im *Open Science Framework* (OSF) präregistriert (osf.io/jsv9z). Für das Gesamtprojekt wurden Apps in deutscher und englischer Sprache identifiziert und auf Ein- und Ausschlusskriterien geprüft. Im vorliegenden Artikel werden die Ergebnisse für alle Apps in deutscher Sprache berichtet.

### Systematische Suche

Die Suche erfolgte für Android-Apps über das Webinterface des deutschen Google Play Store, für iOS Apps über den Apple App Store (iTunes). Folgende Suchbegriffe wurden verwendet: „tinnitus“, „ear ringing“, „ear noise“, „ear buzzing“, „Ohrenklingeln“, „Ohrgeräusch“, „Ohrensausen“. Die Suche fand von November 2020 bis April 2021 statt. Wurde eine App über beide Stores angeboten, so wurde die iOS-Version einbezogen.

### Ein- und Ausschlusskriterien

In einem ersten Schritt wurden die identifizierten Apps anhand von Informationen im Store (Titel, Beschreibung, Nutzerkommentare und Bilder) gescreent. Einschlusskriterien waren: a) für Tinnitus entwickelt und b) in deutscher oder englischer Sprache verfügbar. Apps wurden ausgeschlossen, wenn sie eines der folgenden Kriterien erfüllten: I) App-Bundles (Gruppe von Apps, die gebündelt angeboten werden), II) nicht mehr im Store verfügbar, III) Apps mit eingeschränkten Funktionen, Vollversion bereits in die Analyse eingeschlossen. In einem zweiten Schritt wurden die verbleibenden Apps heruntergeladen und erneut auf Einschlusskriterien a) und b) geprüft. Apps wurden ausgeschlossen, wenn sie eines oder mehrere der Ausschlusskriterien I–III oder eines der folgenden Kriterien erfüllten: IV) technisch nicht hinreichend funktionsfähig, V) in Entwicklungs- oder Testphase, VI) bietet ausschließlich E‑Book oder Artikel zu Tinnitus an, VII) dient der Begleitung einer apparativen Therapie, VIII) Erwerb über Store nicht möglich, IX) kein Zugang zur App.

### Instrumente

Zur Qualitätsanalyse der Apps wurde die deutsche Version der Mobile App Rating Scale (MARS-German; [21]) eingesetzt. Im Rahmen der Studie wurden folgende Subskalen erfasst:

- *Engagement* (A, 5 Items: Unterhaltung; Interesse; individuelle Anpassbarkeit; Interaktivität; Zielgruppe),
- *Funktionalität* (B, 4 Items: Leistung; Usability; Navigation; motorisches, gestisches Design),
- *Ästhetik* (C, 3 Items: Layout; Grafik; visueller Anreiz),
- *Information* (D, 7 Items: Genauigkeit der App-Beschreibung aus dem App-Store; Ziele; Qualität der Information; Quantität der Informationen; visuelle Informationen; Glaubwürdigkeit; Evidenzbasierung) sowie
- *Psychotherapie* (PT, 4 Items: Gewinn für Patienten; Gewinn für Therapeuten; mögliche Risiken, Nebenwirkungen und schädliche Effekte; Übertragbarkeit in die Routineversorgung) und
- *subjektive Qualität* (E, 4 Items: Weiterempfehlung; Häufigkeit der Nutzung; Erwerb der App; Gesamtbewertung).

Für die Bewertung der Items wird eine 5‑stufige Skala genutzt (1 – inadäquat; 2 – schlecht; 3 – akzeptabel; 4 – gut; 5 – exzellent). Der Gesamtwert der Skala (Grundlage Subskalen A–D) sowie die Subskalenwerte dienen als Indikatoren für die App-Qualität (Wertebereich 1–5, mit höheren Werten für eine höhere App-Qualität). Allgemeine Charakteristika der Apps wurden über die erste Sektion der MARS‑G (adaptiert, Vergleich Tab. 1) erhoben.

**Table 1:** 

	*n*
*Plattform*	
iOS	16
Android	5
*Kosten*^a^	
Kostenfrei nutzbar	16
„In-App-Käufe“ verfügbar (Range: 1,99–67,99 €/Monat)	7
Kostenpflichtig (0,69 €/3 Monate–17,99 €/Monat)	5
*Angliederung*	
Unbekannt	4
Gewerblich	11
Regierung	–
Gemeinnützige Organisation	–
Universität	–
Krankenkasse	–
Klinik	–
Andere: Privatperson	3
Andere: Stiftung	3
*Altersgruppe*	
< 18 Jahre	–
18 Jahre+	1
USK „ab 0 Jahren“	5
FSK	–
Andere: Altersangabe Apple^b^	16
*Kategorien im App-Store*	
Lifestyle	–
Medizin	14
Gesundheit und Fitness	6
Andere: Musik und Audio	1
*Technische Aspekte der App*	
Austausch mit anderen (z. B. über soziale Medien)	–
Verfügt über App-Gemeinschaft	–
Benötigt Internetzugang	8
*Einbettung in therapeutische Angebote*	
Keine	18^c^
Kommunikation mit Behandlern über App/Webinterface möglich	–
Zuteilung von Modulen durch Behandler möglich	–
Teilen von Inhalten (z. B. Nutzerstatistiken) mit Behandler möglich	1
Andere: vornehmlich im Studienkontext nutzbar	2
*Einsatzbereich*	
Prävention	–
Behandlung	20
Rehabilitation	–
Nachsorge	–
Andere: Tracking der Symptomatik	1
*Behandlungsart*	
Stand-alone	21
Blended Care (BC)	–
Nicht anwendbar	–
*Unterstützung*	
Guided synchron	–
Guided asynchron	–
Technically guided	2
Unguided	19
*Zertifizierung*	
Keine	17^d^
Entspricht Medizinproduktegesetz	2
Andere: CE-Zertifizierung	4
*Sicherheit und Datenschutz*	
Erlaubt Passwortnutzung	5
Erfordert Login	5
Verfügt über eine Datenschutzerklärung	15
*Barrierefreiheit*	
Ja	–
Nein	21

Allgemeine Charakteristika anhand der MARS-G-Kategorien (adaptiert) für *n* = 21 untersuchte Apps

*USK* Unterhaltungssoftware Selbstkontrolle, *FSK* Freiwillige Selbstkontrolle der Filmwirtschaft

^a^Für 2 Apps ist eine Kostenübernahme durch die Krankenkasse möglich

^b^Davon „Apple 4+“ (*n* = 7), „Apple 12+“ (*n* = 5) und „Apple 17+“ (*n* = 4)

^c^Optionales Angebot einer begleiteten Nutzung für eine App

^d^Eine App wurde nach IEC-Normen und GAMP 5 erstellt

Zudem wurde von den Autor:innen (AR, DL, CW) ein Katalog von im Tinnitusbereich üblichen Interventionselementen erstellt. Dieser wurde auf Basis von Studien zu Elementen von Interventionen [26], der S3-Leitlinie Chronischer Tinnitus [27], der Leitlinie evidenzbasierte Gesundheitsinformation [28], von Übersichtsarbeiten zu Tinnitus [29–31] und einem Therapiemanual [32] erstellt. Der Katalog diente als Grundlage, um die App-Inhalte zu katalogisieren. Zudem wurde für jede App bewertet, welche der gefundenen Interventionselemente zentral bedeutsam sind.

Zur Erfassung der eingesetzten BCTs kam die Behavior Change Technique Taxonomy („v1“; [23]) zum Einsatz. Die Taxonomie umfasst 93 Techniken, die im Rahmen einer Intervention zur Veränderung von Gesundheitsverhalten eingesetzt werden können (z. B. „self-monitoring of behaviour“ oder „action planning“). Diese sind 16 Kategorien (z. B. „feedback and monitoring“ oder „goals and planning“) zugeordnet.

### Bewertungstraining

Vor Beginn der Analyse der Apps absolvierten 3 Bewerterinnen ein Training. Dieses umfasste: A) ein Trainingsvideo zur MARS‑G [21] sowie einen Ausschnitt des Trainingsvideos mit beispielhaftem Rating zur englischen Originalskala [20]; B) „Practices“ der Online-Plattform zur BCT Taxonomy v1 (alle Bewerterinnen) sowie ein Online-Training (Bewerterin 1; http://www.bct-taxonomy.com/); die Erstellung und Diskussion von Beispielen zur praktischen Umsetzung der 93 BCTs im Rahmen einer App für Tinnitus und C) eine unabhängige Analyse von 5 Trainings-Apps aus dem Themenfeld Schmerz und Diskussion der Ergebnisse.

### Datenerhebung und Analyse

Jede App wurde unabhängig von 2 Bewerterinnen (M. Sc. Psychologie) für 20 min getestet und anschließend bewertet. Getestet wurden jeweils die Vollversionen der Apps. Jede Bewerterin erhielt eine Liste der zu bewertenden Apps mit vorgegebener Reihenfolge. Die Reihenfolge unterschied sich dabei zwischen den Bewerterinnen. Nach Abschluss der Ratings prüfte eine nicht am Projekt beteiligte Person die Ergebnisse der beiden Bewerterinnen zu Items der MARS‑G mit der Antwortoption „not applicable“. Differenzen zwischen den Bewerterinnen dazu, ob Items für die jeweilige App bewertet werden können, wurden diskutiert. Zur Analyse der Übereinstimmung der Bewertungen (Interrater-Reliabilität) für den Gesamtwert der MARS‑G wurde die „Intra-Class-Correlation“ (ICC; „two-way mixed effects, absolute agreement“) berechnet. Fiel die ICC unter 0,75 wurde eine dritte Bewerterin hinzugezogen. Nach Abschluss der Ratings diskutierten beide Bewerterinnen auf Basis der Differenzen die Ergebnisse zu Interventionselementen und BCTs für jede App. Konnte kein Konsens erzielt werden, wurde die dritte Bewerterin einbezogen. Für die Subskalen der MARS‑G sowie den Gesamtwert werden die gemittelten Werte aus beiden Ratings berichtet. Um Evaluationsstudien zu den Apps zu identifizieren, wurden Datenbanken (z. B. Pubmed, PsycInfo) und Systematic Reviews durchsucht und alle Entwickler:innen per E‑Mail kontaktiert. Zur Datenaufbereitung und Analyse wurde die Software IBM SPSS Statistics 28 (IBM Corp., Armonk, NY, USA) [33] eingesetzt.

## Ergebnisse

### Systematische Suche

**Figure 1:**
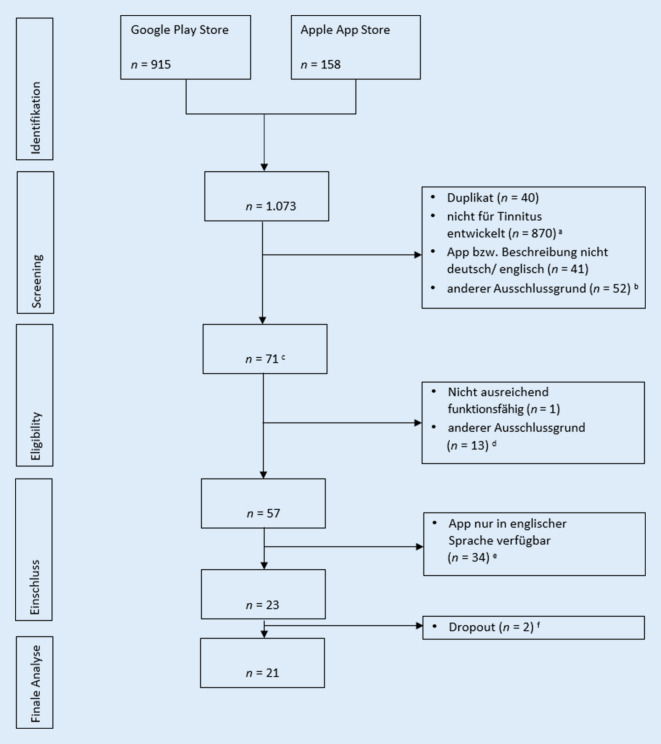

Über die systematische Suche in den Stores wurden *n* = 1073 Apps identifiziert (Google Play Store *n* = 915, Apple App Store *n* = 158). 40 Apps waren in beiden Stores verfügbar. Weitere Apps wurden im ersten Schritt (Screening) ausgeschlossen, da a) sie nicht für Tinnitus entwickelt wurden (*n* = 870), b) App- oder Store-Beschreibung nicht in deutscher oder englischer Sprache vorlag (*n* = 41) oder c) ein sonstiger Ausschlussgrund erfüllt war (z. B. App-Bundles; *n* = 52). Anschließend wurden 71 Apps heruntergeladen und erneut anhand der Kriterien geprüft. Eingeschlossen wurden *n* = 23 deutschsprachige Apps. 2 Apps konnten nicht analysiert werden (nicht mehr im Store bzw. nicht mehr auf Deutsch verfügbar). In Summe wurden 21 Apps analysiert. Details zur Auswahl finden sich im Flussdiagramm in Abb. 1.

### Allgemeine Charakteristika

Über die MARS‑G wurden allgemeine Merkmale der Apps erfasst. Es wurden *n* = 16 Apps mit iOS- und *n* = 5 Apps mit Android-Betriebssystem untersucht. Die Apps stammten meist von gewerblichen Anbieter:innen (*n* = 11), für einzelne Apps waren die Hersteller:innen nicht zu ermitteln (*n* = 4). Für 2 Apps (davon eine gelistet im Verzeichnis für digitale Gesundheitsanwendungen, DiGA^1^) war eine Kostenübernahme durch die Krankenversicherung möglich. Eine App bot das Teilen von Inhalten mit Behandler:innen an, eine wurde optional für eine therapiebegleitende Nutzung angeboten und 2 waren aktuell vornehmlich im Studienkontext nutzbar. Die Apps wurden alleinstehend (stand-alone) zur Behandlung des Tinnitus bzw. zur Behandlung der Belastung durch den Tinnitus (*n* = 20) sowie zum Tracking der Symptomatik (*n* = 1) eingesetzt. Für 2 Apps konnten Nutzende eine technische Unterstützung in Anspruch nehmen. Nach Angaben der Hersteller:innen entsprachen 2 Apps dem Medizinproduktegesetz, 4 waren CE-zertifiziert und eine wurde auf Basis der Normen IEC 62304 [34] bzw. IEC 82304 [35] sowie des Regelwerks „Good Automated Manufacturing Practice 5“ (GAMP 5) erstellt. Tab. 1 zeigt die allgemeinen Charakteristika der Apps.

### Qualität

Im Hauptteil der MARS‑G wird die Qualität der Apps erfasst. Der Gesamtwert für die Qualität lag im durchschnittlichen Bereich (M = 3,37; SD = 0,39, Range 1–5). Am besten bewertet wurde die *Funktionalität* (M = 4,21; SD = 0,45), gefolgt von der *Ästhetik* (M = 3,39; SD = 0,57), den *Informationen* (M = 3,07; SD = 0,50) sowie dem *Engagement* (M = 2,81; SD = 0,68). Niedrigere Werte zeigten sich für die Zusatzskalen *Psychotherapie (*M = 2,06; SD = 0,42) und *subjektive Qualität* der App (M = 2,07; SD = 0,56). Da nur für eine App Evaluationsstudien vorlagen, konnte das Item zur Evidenzbasierung (Item 19, D) nur für diese App bewertet werden. Das Item zu möglichen Risiken, Nebenwirkungen und schädlichen Effekten (Item 22, PT) wurde für 4 Apps von beiden Bewerterinnen mit einem Wert kleiner 3 bewertet. Gründe hierfür waren zum Beispiel: angegebene Evidenz entsprach nicht der aktuellen Studienlage, Instruktionen zur App-Nutzung bzw. zur Durchführung von Übungen waren unklar oder unvollständig, das Ziel der App bzw. einzelner Übungen war nicht eindeutig erkennbar. Die Übereinstimmung der Bewertungen war ausreichend (ICC 0,76, KI 95 % 0,38–0,91). Tab. 2 zeigt die Mittelwerte für alle Subskalen der MARS‑G sowie den Gesamtwert für die Apps.

**Table 2:** 

App-Name	MARS‑G Gesamt	Engagement	Funktionalität	Ästhetik	Information	Psychotherapie	Subjektive Qualität
Beltone Tinnitus Calmer^b, c^	4,02	3,40	4,75	4,17	3,75	2,17	3,00
ReSound Tinnitus Relief^b, c^	3,92	3,50	4,75	4,00	3,42	3,17	2,75
Tinnitracks Tinnitus Therapie^b, c^	3,91	4,40	3,75	3,67	3,83	2,50	2,88
UNITI Tinnitus Studie^b, c^	3,86	3,20	4,50	4,33	3,42	2,50	3,00
Kalmeda Tinnitus-App^b, c^	3,75	4,30	3,88	4,00	2,83	2,17	2,50
Widex Zen. Tinnitus Management^b, c^	3,69	2,80	4,75	4,00	3,20	2,17	2,38
Track Your Tinnitus^b, c^	3,60	2,90	4,50	3,33	3,67	2,33	2,00
TINNITUS, WHITE NOISE, SCHLAF^b^	3,41	2,30	4,50	3,83	3,00	2,33	2,63
Shades of Noise^b, c^	3,40	3,00	3,88	3,33	3,38	2,00	1,88
Dr Mollin Tinnitus^b, c, d^	3,30	2,30	4,50	4,00	2,40	1,67	2,00
Relax Noise 3 – Tinnitus^a^	3,29	2,20	4,63	2,83	3,50	2,17	1,63
Tonal Tinnitus Therapy^a^	3,28	2,90	4,38	3,17	2,67	1,50	1,63
Tinnitus HQ^b^	3,28	2,90	3,88	3,33	3,00	1,83	1,88
Starkey Relax^b, c^	3,26	2,80	3,88	3,17	3,20	2,00	2,13
Tinnitus Notched Tunes^a^	3,22	2,80	3,63	3,33	3,13	2,00	2,00
TinniTrain^b, c^	3,21	2,60	3,75	3,00	3,50	2,17	2,13
Tinnitus Masker^b, c, d^	3,20	2,60	4,38	3,33	2,50	1,67	1,50
White Noise for Sleep. Generator for Tinnitus^a^	3,00	2,20	4,63	2,67	2,50	2,00	1,75
Tinnitus help^b, c, d^	2,81	2,30	3,13	2,83	3,00	1,67	1,25
Tinnitus Relief App. Klangtherapie^a^	2,78	2,00	4,13	2,50	2,50	2,17	1,63
Tinnitus DE^b, c, d^	2,60	1,70	4,38	2,33	2,00	1,17	1,00
Gesamt M (SD)	3,37 (0,39)	2,81 (0,68)	4,21 (0,45)	3,39 (0,57)	3,07 (0,50)	2,06 (0,42)	2,07 (0,56)

Mittelwerte M der Ratings von Bewerterinnen 1 und 2 für die Subskalen und den Gesamtwert der MARS‑G [21]

^a^Getestet für Android

^b^Getestet für iOS

^c^App war für beide Betriebssysteme verfügbar, Duplikate wurden anhand von Informationen im App-Store (z. B. Angaben zu Entwickler:innen, Name der App, Bilder oder App-Beschreibung) identifiziert

^d^Item 22, Subskala PT von beiden Bewerterinnen mit einem Wert kleiner 3 bewertet. Gesamt = mittlere Werte über alle Apps

### Interventionselemente

In einem weiteren Schritt wurde untersucht, welche Interventionselemente bei Tinnitus (z. B. Psychoedukation, Aufmerksamkeitslenkung) die Apps anbieten und ob diese im Fokus der Nutzung stehen. Am häufigsten wurden den Nutzenden Geräusche angeboten (*n* = 18 Apps; z. B. verschiedene Arten Rauschen, Naturklänge), was ebenfalls am häufigsten als zentrales Element identifiziert wurde (*n* = 15). Außerdem fanden sich oft Informationen zum Ziel der App (*n* = 15) sowie zum Preis (Kosten, *n* = 21). Es wurden Charakteristika des Ohrgeräusches erfasst (*n* = 13) oder Informationen zu Tinnitus vermittelt (*n* = 9), 2 Interventionselemente, die ebenfalls häufig im Fokus der App standen (*n* = 8 bzw. *n* = 5). Nur sehr wenige Apps boten Achtsamkeitsübungen (*n* = 3), frequenzmodulierte Musiktherapie (*n* = 3) und den Aufbau positiver Aktivitäten (*n* = 3) an. Die Zahl der Interventionselemente unterschied sich mit 2 bis 20 Elementen pro App deutlich voneinander. 5 Apps enthielten 11 oder mehr Interventionselemente, 15 Apps nur 2 bis maximal 7. Insgesamt wurden 31 unterschiedliche Interventionselemente identifiziert. Tab. 3 gibt einen Überblick zu den Elementen der Apps. Eine Übersicht zu den identifizierten Interventionselementen für jede App findet sich im Onlinematerial 3.

**Table 3:** 

Interventionselement	Element in *n *Apps enthalten	Element für *n* Apps zentral
*Erfassung von Tinnituscharakteristika*	13	8
*Erfassung der Belastung durch den Tinnitus*	7	2
*Analyse von Risikofaktoren für die Tinnitusentstehung*	8	–
Informationsvermittlung	7	1
Erfassung	1	–
*Analyse von Einflussfaktoren auf die Entstehung und Aufrechterhaltung der Tinnitusbelastung*	4	–
Informationsvermittlung	4	1
Erfassung	1	–
*Informationen zu/Prüfung des Vorliegens von weiteren otologischen Symptomen (z.* *B. Hörminderung, Hyperakusis)*	5	–
*Informationen zur Intervention in Bezug auf*		
Ziel der Maßnahme	15	–
Prognose bei Nichtintervention	–	–
Behandlungsoptionen (inkl. Nichtbehandlung)	–	–
Informationen zu Unsicherheiten und zu fehlender Evidenz	1	–
Wahrscheinlichkeiten für Erfolg, Misserfolg und Nebenwirkungen der Maßnahme und der Behandlungsoptionen	–	–
Kosten	21	–
*Informationen zu Tinnitus/Psychoedukation*	9	5
*Entspannungsverfahren*	5	4
*Achtsamkeitsübungen*	3	1
*Identifikation von dysfunktionalen Gedanken*	4	1
*Veränderung von dysfunktionalen Gedanken*	4	1
*Aufmerksamkeitslenkung*	1	–
*Konfrontation mit dem Tinnitus*	1	–
*Expositionsübungen zu vermiedenen Geräuschumgebungen oder Tätigkeiten*	–	–
*Emotionsregulation*	1	1
*Soziale Unterstützung (z.* *B. Kontaktmöglichkeiten zu anderen Betroffenen)*	1	–
*Förderung der sozialen Kompetenz*	–	–
*Vermittlung von Hörtaktiken bei Hörminderung*	1	–
*Techniken zur Verbesserung des Schlafs*	6	–
*Techniken zur Verbesserung von Konzentration und Arbeitseffizienz*	1	–
*Einsatz von Geräuschen (z.* *B. Rauschen, Naturklänge, Musik)*	18	15
*Auditorisches Diskriminationstraining*	1	1
*Frequenzmodulierte Musiktherapie*	3	3
*Akustische Neuromodulation*	1	1
*Biofeedback*	–	–
*Neurofeedback*	–	–
*Rückfallprävention*	1	–
*Sonstige *^*a*^ *(z.* *B. Aufbau positiver Aktivitäten)*	10	–

^a^ Aufschlüsselung der sonstigen Interventionselemente siehe Onlinematerial 2

### Techniken der Verhaltensänderung (BCTs)

Die Apps wurden außerdem daraufhin untersucht, welche der 93 BCTs nach Michie [23] identifiziert werden können. Insgesamt wurden 69 der 93 BCTs in keiner der untersuchten Apps eingesetzt, nur 24 der 93 BCTs wurden mindestens einmal identifiziert. Am häufigsten wurde die Technik „feedback on behaviour“ („Feedback zum Verhalten“) eingesetzt (*n* = 9), gefolgt von „instruction on how to perform the behaviour“ („Anleitung zur Durchführung des Verhaltens“; *n* = 6) sowie „behavioral practice/rehearsal“ („Verhaltensweisen üben/einüben“; *n* = 6) und „prompts/cues“ („Aufforderungen/Hinweise“; *n* = 6). Die Anzahl der eingesetzten BCTs unterschied sich erheblich: 9 Apps setzten keine BCTs ein, während 2 Apps eine große Anzahl an verschiedenen BCTs nutzten (10 bzw. 18 BCTs). Tab. 4 zeigt eine Übersicht zu allen identifizierten BCTs. Im Onlinematerial 4 werden die für die einzelnen Apps identifizierten BCTs abgebildet.

**Table 4:** 

Kategorie	Behavior Change Technique (BCT)	BCT in *n* Apps enthalten
1. Goals and planning (5/9)^a^	1.1 Goal setting (behaviour)	3
	1.2 Problem solving	1
	1.4 Action planning	1
	1.6 Discrepancy between current behavior and goal	1
	1.9 Commitment	1
2. Feedback and monitoring (3/7)	2.2 Feedback on behaviour ^b^	9
	2.4 Self-monitoring of outcome(s) of behaviour	2
	2.7 Feedback on outcome(s) of behaviour	1
3. Social support (1/3)	3.1 Social support (unspecified)	3
4. Shaping knowledge (2/4)	4.1 Instruction on how to perform the behavior ^c^	6
	4.3 Re-attribution	1
5. Natural consequences (2/6)	5.4 Monitoring of emotional consequences	1
	5.6 Information about emotional consequences	5
6. Comparison of behaviour (0/3)	–	0
7. Associations (3/8)	7.1 Prompts/cues	6
	7.7 Exposure	1
	7.8 Associative learning	1
8. Repetition and substitution (1/7)	8.1 Behavioral practice/rehearsal	6
9. Comparison of outcomes (0/3)	–	0
10. Reward and threat (3/11)	10.3 Non-specific reward	1
	10.6 Non-specific incentive	1
	10.9 Self-reward	1
11. Regulation (1/4)	11.2 Reduce negative emotions	1
12. Antecedents (0/6)	–	0
13. Identity (2/5)	13.2 Framing/reframing	4
	13.4 Valued self-identity	1
14. Scheduled consequences (0/10)	–	0
15. Self-belief (1/4)	15.3 Focus on past success	1
16. Covert learning (0/3)	–	0

BCTs für *n* = 21 untersuchte Apps. Einsatz der Behavior Change Technique Taxonomy v1 nach Michie et al. [23]

^a^Aus Gründen der Lesebarkeit sind nur BCTs aufgeführt, die identifiziert werden konnten. Die zweite Zahl beschreibt die Gesamtanzahl der BCTs in der entsprechenden Kategorie

^b^z. B. Rückmeldung an Nutzende, wie viele Minuten pro Woche Atemübungen durchgeführt wurden

^c^z. B. Anleitung zur Durchführung Progressive Muskelentspannung

## Diskussion

Dies ist die erste Studie, die explizit deutschsprachige Tinnitus-Apps hinsichtlich Qualität, eingesetzter Interventionselemente und BCTs untersucht. Insgesamt konnten 21 Apps analysiert werden.

### Qualität der Apps.

Die untersuchten Apps wiesen eine durchschnittliche Qualität entsprechend der MARS‑G [21] auf. Am höchsten bewertet wurde die *Funktionalität* der Apps. Die niedrigsten Werte erhielten die Zusatzskalen *subjektive Qualität* und *Psychotherapie*. Mit Blick auf die Interventionselemente wurden am häufigsten Geräusche verschiedener Art angeboten, Charakteristika der Ohrgeräusche erfragt oder Informationen vermittelt. Typische Interventionen der Tinnitusbehandlung (z. B. Aufmerksamkeitslenkung, Entspannungsübungen) fanden sich überraschend selten. Die Apps unterschieden sich zudem stark in der Zahl der eingesetzten BCTs. Während 2 Apps verschiedenste Techniken umsetzten, konnten bei der Mehrzahl keine oder nur sehr wenige BCTs identifiziert werden.

In unserer Studie war die Varianz in der Qualität der Apps, entsprechend der MARS‑G, auffällig. Während die *Funktionalität* der Tinnitus-Apps gut bewertet wurde, gibt es hinsichtlich der Qualität der *Informationen* sowie des Ausmaßes, in dem die App das *Engagement* der Nutzenden fördert, deutliches Verbesserungspotenzial. Dieses Ergebnis deckt sich mit Untersuchungen zu Apps bei Depression oder posttraumatischer Belastungsstörung (PTBS), auch hier wurde die Funktionalität der Apps am höchsten bewertet [36, 37]. Auch das Gesamtergebnis hinsichtlich der schwankenden Qualität spiegelt die Ergebnisse früherer Arbeiten sowohl zu Tinnitus-Apps [16] als auch zu Apps aus anderen Themenbereichen [36–39] wider. Die ständig steigende Anzahl von Apps in den Stores bei gleichzeitig schwankender Qualität erschwert es Betroffenen und Behandelnden, eine qualitativ hochwertige App auszuwählen. Eine öffentliche Datenbank, die reliable, valide und standardisierte Expert:innenratings (z. B. anhand der MARS-G) zugänglich macht, könnte zur informierten Entscheidung für eine App bei spezifischen Erkrankungen oder Einsatzbereichen beitragen [21]. Auf bestehenden Plattformen wie „PsyberGuide“ [7] oder „Mobile Health App Database (mHAD) Germany“ ist es aktuell jedoch leider nicht möglich, gezielt nach Apps zu suchen, die für Tinnitus entwickelt wurden. Im DiGA-Verzeichnis finden sich bisher (Stand: Juli 2023) lediglich 2 Apps zum Einsatz bei Tinnitus.

Die Auswahl einer geeigneten App wird darüber hinaus durch die meist fehlenden wissenschaftlichen Wirksamkeitsuntersuchungen der Tinnitus-Apps erschwert. Für lediglich eine App lagen Publikationen zur wissenschaftlichen Evaluation vor, wobei es sich dabei um unkontrollierte Studien mit kleinen Stichprobengrößen handelt [40]. Für eine weitere App wurde eine Evaluation durch ärztliche Behandelnde berichtet, die zur Entwicklung der Symptomatik der Patient:innen nach der App-Nutzung befragt wurden [41]. In der Zwischenzeit wurde eine weitere Studie zur Evaluation einer App veröffentlicht [42]. Das Ergebnis unserer Studie, dass es für eine substanzielle Anzahl von Apps keine wissenschaftliche Evidenz gibt, bestätigen ebenfalls frühere Übersichtsarbeiten zu Tinnitus-Apps [43, 44] sowie zu Apps aus anderen Themenfeldern (z. B. PTBS, Depression, Schlaf, Achtsamkeit; [36–38, 45]).

Im Qualitätsrating wurden einzelne Übungen identifiziert, die bei selbstständiger Durchführung ohne zusätzliche Instruktionen negative Effekte auslösen könnten (z. B. bei unzureichender Anleitung zu einem Hörtraining bei Hyperakusis oder zur Exposition gegenüber dem Tinnitus). Zudem entsprach die Einordnung zur Evidenz des angebotenen Verfahrens teilweise nicht der aktuellen Studienlage. Auch dieses Ergebnis ist konsistent mit früheren Studien, die zeigten, dass Inhalte der Tinnitus-Apps für Betroffene potenziell negative Effekte haben könnten [13]. Sereda und Kollegen [8] summierten, dass manche Inhalte von Tinnitus-Apps, wenn sie ohne Begleitung durch geschulte Therapeut:innen eingesetzt werden, nicht für Tinnitusbetroffene geeignet sein könnten (z. B. Übungen zur Identifikation maladaptiver Gedanken). Nach unserem Kenntnisstand untersuchte bisher nur eine Studie negative Effekte bzw. mögliche Risiken einer App-Nutzung. Schlee et al. (2016) summierten, dass die regelmäßige Nutzung einer App zum Tracking der Tinnitussymptomatik keine bedeutsamen negativen Effekte auf die wahrgenommene Lautstärke des Tinnitus oder die Belastung durch das Ohrgeräusch hatte [46].

### Verwendete Interventionselemente.

Neben der Qualität interessierte uns auch, welche Interventionselemente in den Apps genutzt werden. Auch hier deckt sich unser Ergebnis, dass primär Geräusche (z. B. Naturgeräusche oder Rauschen) eingesetzt werden, mit den Ergebnissen anderer Autor:innen [13, 16]. Mögliche Gründe sind die vergleichsweise leichte Umsetzbarkeit per App sowie, dass Geräusche von Betroffenen gern genutzt werden [8]. Von wissenschaftlicher Seite fehlt es an Evidenz für den alleinigen Einsatz von Geräuschen bei chronischem Tinnitus [31, 47], vielmehr müssen auch mögliche Risiken durch das (dauerhafte) Maskieren des Tinnitus berücksichtigt werden (z. B. fehlende Habituation; [29, 32]). Weitere häufige Interventionselemente waren Informationsvermittlung zum Tinnitus oder die Erfassung von Tinnituscharakteristika (z. B. Frequenz). Auch diese Elemente sind vergleichsweise leicht per App umsetzbar und können von Betroffenen ohne viel Anleitung genutzt werden. Demgegenüber waren zentrale Elemente der KVT (z. B. Identifikation und Veränderung von dysfunktionalen Gedanken, Aufmerksamkeitslenkungsübungen, Entspannungstraining) deutlich seltener oder gar nicht integriert. Da es gerade für die KVT bei Tinnitus sehr gute Evidenzbelege gibt [48, 49], erscheint eine stärkere Integration dieser Elemente als notwendiger nächster Schritt für wirksame Tinnitus-Apps. Gleichzeitig könnte für solche Apps eine umfangreichere Anleitung nötig sein, damit einerseits das Potenzial der Übungen ausgeschöpft werden kann, anderseits aber auch potenziell negative Effekte vermieden werden.

### Techniken der Verhaltensänderung (BCTs).

Dabei könnte auch die Analyse von Apps entlang der Behavior-Change-Taxonomie dazu beitragen, wichtige Bestandteile („active ingredients“) zu identifizieren und gezielt umzusetzen [23]. In der vorliegenden Studie zeigte sich, dass solche BCTs in der überwiegenden Zahl der Apps noch sehr wenig eingesetzt werden. Daher muss vermutet werden, dass Betroffene durch die App-Nutzung nicht ausreichend aktiviert werden und daher Strategien zur Tinnitusbewältigung weniger gut erlangen. Mit der systematischen Identifikation und Beschreibung der in Apps eingesetzten BCTs führt die vorliegende Studie neuere Entwicklungen fort, die sich bislang auf BCTs im Rahmen von browserbasierten Interventionen bzw. verschiedenen Selbsthilfeinterventionen konzentrierten [50–52].

### Limitationen und Implikationen

Die systematische Suche wurde im April 2021 abgeschlossen. Es ist möglich, dass zwischenzeitlich neue Apps entwickelt wurden, von uns untersuchte Apps nicht mehr im Store verfügbar oder verändert sind. Zudem könnte es sein, dass weitere Apps wissenschaftlich evaluiert worden sind. Die in unserer Studie dargestellten und entwickelten Kriterien bieten jedoch eine gute Grundlage für regelmäßige Updates, durch die die Veränderungen in diesem dynamischen Feld besser abgebildet werden können. Als weitere Limitation ist zu nennen, dass für die Suche ausschließlich die beiden größten App Stores genutzt wurden. Nicht einbezogen wurden weitere Stores (z. B. Microsoft) oder App Libraries (z. B. NHS Apps Library). Die dargestellten Ergebnisse beziehen sich ausschließlich auf Apps in deutscher Sprache und sind nur eingeschränkt generalisierbar. Es kann zudem kein Rückschluss gezogen werden, wie gut die Elemente umgesetzt sind, da lediglich das Vorliegen der Elemente kodiert wurde. Zudem sollte in zukünftigen Untersuchungen evaluiert werden, welche Interventionselemente und BCTs Betroffene wirksam im Tinnitusmanagement unterstützen können.

Aus der multiperspektivischen Analyse ergibt sich insgesamt ein Bild, dass Apps für Tinnitus bislang häufig eher für eine passive Nutzung konzipiert sind, d. h., dass die Nutzenden wenig dazu motiviert werden, selbst aktiv zu werden. So fanden sich bei der Untersuchung mittels MARS niedrige Werte für die Förderung des Engagements der Betroffenen. Des Weiteren wurde als zentrales Interventionselement die Nutzung von Geräuschen identifiziert, bei der sich Betroffene passiv einem Reiz aussetzen. Aktive Strategien zur Änderung von Gesundheitsverhalten werden seltener integriert, wie die Analyse der BCTs deutlich macht. Entsprechend könnte das Potenzial von Apps deutlich besser genutzt werden, indem aktives Tinnitusmanagement inhaltlich breiter in Apps integriert wird (z. B. Übungen zur Aufmerksamkeitslenkung; Übungen zur Exposition gegenüber dem Tinnitus) und dazu bewährte Techniken zur Veränderung dieses Gesundheitsverhaltens digital umgesetzt werden.

Die niedrigen Werte in *subjektiver Qualität* und *Psychotherapie* der MARS‑G weisen darauf hin, dass insbesondere die frei verfügbaren Apps nicht klar empfohlen werden können und einzelne Interventionselemente, bei einseitiger Nutzung in Selbsthilfe, sogar negative Effekte erwarten lassen. Entsprechend sollten behandelnde Ärzt:innen oder Psychotherapeut:innen die Nutzung von Tinnitus-Apps bei ihren Patient:innen aktiv erfragen und die Interventionselemente der jeweiligen App professionell einordnen. Die Motivation von Betroffenen zur Nutzung einer App sollte als Ressource verstanden werden. Entsprechend sollten Betroffene ermutigt werden hilfreiche Funktionen der Apps (weiter) zu nutzen, dies als Teil des Tinnitusmanagements zu verstehen und sie gleichzeitig in einen übergreifenden Präventions- oder Behandlungsplan zu integrieren. Voraussetzung dafür sind niederschwellige Informationsquellen für Behandelnde.

## Fazit

Dies ist die erste Übersichtsarbeit zu deutschsprachigen Tinnitus-Apps mit einer Analyse der Qualität sowie der eingesetzten Interventionselemente und BCTs. Trotz der hohen Zahl der verfügbaren Apps wird das Potenzial, mit der App eine echte Intervention anzubieten, bisher nicht ausgeschöpft. Die Mehrzahl der Apps nutzt nur wenige Interventionselemente und weitere Elemente der KVT, die sich in vorherigen Studien bewährt haben, kommen nur selten bis gar nicht zum Einsatz. Die fehlende wissenschaftliche Evaluation von Tinnitus-Apps erschwert es Betroffenen und Behandelnden eine qualitativ hochwertige und wirksame App auszuwählen. Zukünftige Forschung sollte daher gezielt typische Interventionselemente der Tinnitusbehandlung integrieren. Die Taxonomie der BCTs könnte dabei als Anregung zur Gestaltung von Apps zum aktiven Tinnitusmanagement genutzt werden. Die multiperspektivische Evaluation kann als Muster für Apps zu anderen chronischen Erkrankungen dienen.

### Supplementary Information





## Footnotes

1 DiGA-Verzeichnis: https://diga.bfarm.de/de/verzeichnis (Zugriff: 19.05.2023).
