# Supplementary material for: Apps for tinnitus? A systematic review on quality, intervention components, and behavior change techniques
Source: Bundesgesundheitsblatt Gesundheitsforschung Gesundheitsschutz. 2023 Dec 12;67(2):203–14. [Article in German] doi: 10.1007/s00103-023-03805-1 (PMC10834554; doi:10.1007/s00103-023-03805-1)
Supplement: Supplementary file 1 [file 103_2023_3805_MOESM1_ESM.pdf]

Zusätzliches Onlinematerial zum Beitrag:

„Mit Apps gegen den Tinnitus? - Ein systematisches Review zu Qualität, Interventionselementen und Techniken der Verhaltensänderung“

von Rinn et al.

(Bundesgesundheitsblatt, Heft 2/2024)

Onlinematerial 1: Aufschlüsselung Screening-Kriterium a) zur Auswahl der Tinnitus-Apps

App nicht für Tinnitus entwickelt: Davon *n* = 112 Apps mit Geräuschen bei Schlaflosigkeit, zur Förderung der Konzentration, zur Begleitung von Meditation / Yoga, zur Stressreduktion, bei Tinnitus; *n* = 17 Apps zur Anpassung eines Hörgerätes, bietet Tinnitus-Signal oder –Programm; *n* = 19 Apps, bei denen Tinnitus im Titel oder in der Beschreibung erwähnt wird (z.B. Informationen zu verschiedenen HNO-Erkrankungen, Frequenzgeneratoren, Anhören eines Tinnitus-Tons).

Onlinematerial 2: Aufschlüsselung der sonstigen Interventionselemente bei Tinnitus

Aufbau positiver Aktivitäten/ Verhaltensaktivierung (*n* = 3); Stressmanagement-Training (*n* = 1); Übungen aus Hörtherapie (Schulung des Richtungshörens, der Hörwahrnehmung, *n* = 1); Abfrage von/ Fokus auf eigene Stärken und Erfolge (*n* = 1, als zentral eingeordnet); Förderung von Akzeptanz (*n* = 1, als zentral eingeordnet); Erfassung von Motiven/Bedürfnissen und Ableitung von Lebenszielen (*n* = 1); Tinnituszentrierte Musiktherapie nach Cramer (TIM, laut zugehöriger Website) (*n* = 1, als zentral eingeordnet); Tracking von z.B. Stimmung, Stress, Konzentration (zusätzlich zu Erfassung von Tinnitus-Charakteristika sowie Belastung; *n* = 1, als zentral eingeordnet).

Onlinematerial 3: Interventionselemente aufgeschlüsselt für die einzelnen Apps.

Interventionselement	Relax Noise 3 – Tinnitus	Dr Mollin Tinnitus	Shades of Noise	TinniTrain	Tinnitus Notched Tunes	Tinnitus Relief App. Klangtherapie	White Noise for Sleep. Generator for Tinnitus	Tonal Tinnitus Therapy	Tinnitracks Tinnitus Therapie	ReSound Tinnitus Relief	Tinnitus DE	Widex Zen. Tinnitus Management	Kalmeda Tinnitus-App	tinnitus help	Tinnitus HQ	Track Your Tinnitus	Beltone Tinnitus Calmer	Starkey Relax	Tinnitus Masker	TINNITUS, WHITE NOISE, SCHLAF	UNITI Tinnitus Studie
Informationen zu Tinnitus/ Psychoedukation		✓							✓	✓	✓	✓	✓				✓	✓			✓
Analyse von Risikofaktoren für die Tinnitusentstehung									✓	✓		✓	✓			✓	✓	✓			✓
Analyse von Einflussfaktoren auf die Entstehung und Aufrechterhaltung der Tinnitusbelastung									✓			✓	✓								✓
Informationen zu/ Prüfung des Vorliegens von weiteren otologischen Symptomen									✓	✓			✓				✓				✓
Erfassung von Tinnitus-Charakteristika			✓	✓	✓			✓	✓	✓			✓	✓	✓	✓	✓		✓		✓
Erfassung der Belastung durch den Tinnitus			✓						✓	✓			✓			✓	✓				✓
Informationen zur Intervention in Bezug auf																					
Ziel der Maßnahme	✓	✓			✓			✓	✓	✓	✓	✓	✓	✓	✓	✓	✓	✓			✓
Informationen zu Unsicherheiten und zu fehlender Evidenz								✓													
Kosten	✓	✓	✓	✓	✓	✓	✓	✓	✓	✓	✓	✓	✓	✓	✓	✓	✓	✓	✓	✓	✓
Entspannungsverfahren									✓	✓		✓	✓				✓				
Achtsamkeitsübungen										✓			✓				✓				
Identifikation von dysfunktionalen Gedanken									✓	✓			✓				✓				
Veränderung von dysfunktionalen Gedanken									✓	✓			✓				✓				
Aufmerksamkeitslenkung									✓												
Konfrontation mit dem Tinnitus													✓								

Interventionselemente aufgeschlüsselt für *n* = 21 untersuchte Apps. Aus Gründen der Lesbarkeit sind nur Elemente aufgeführt, die für mindestens eine App identifiziert wurden.

Fortsetzung **Onlinematerial 3**: Interventionselemente aufgeschlüsselt für die einzelnen Apps.

Interventionselement	Relax Noise 3 – Tinnitus	Dr Mollin Tinnitus	Shades of Noise	TinniTrain	Tinnitus Notched Tunes	Tinnitus Relief App. Klangtherapie	White Noise for Sleep. Generator for Tinnitus	Tonal Tinnitus Therapy	Tinnitracks Tinnitus Therapie	ReSound Tinnitus Relief	Tinnitus DE	Widex Zen. Tinnitus Management	Kalmeda Tinnitus-App	tinnitus help	Tinnitus HQ	Track Your Tinnitus	Beltone Tinnitus Calmer	Starkey Relax	Tinnitus Masker	TINNITUS, WHITE NOISE, SCHLAF	UNITI Tinnitus Studie
Emotionsregulation													✓								
Soziale Unterstützung													✓								
Vermittlung von Hörtaktiken bei Hörminderung									✓												
Techniken zur Verbesserung des Schlafs									✓	✓		✓	✓				✓				✓
Techniken zur Verbesserung von Konzentration und Arbeitseffizienz																					✓
Einsatz von Geräuschen	✓	✓	✓		✓	✓	✓	✓		✓	✓	✓	✓	✓	✓		✓	✓	✓	✓	✓
Auditorisches Diskriminationstraining				✓																	
Frequenzmodulierte Musiktherapie					✓				✓						✓						
Akustische Neuromodulation								✓													
Rückfallprävention									✓												
Aufbau positiver Aktivitäten										✓			✓				✓				
Stressmanagement-Training									✓												
Übungen aus Hörtherapie																					✓
Abfrage von/ Fokus auf eigene Stärken und Erfolge													✓								
Förderung von Akzeptanz													✓								
Erfassung von Motiven/Bedürfnissen und Ableitung von Lebenszielen													✓								
Tinnituszentrierte Musiktherapie Cramer														✓							
Tracking Stimmung, Stress, Konzentration																✓					

Interventionselemente aufgeschlüsselt für $n = 21$ untersuchte Apps. Aus Gründen der Lesbarkeit sind nur Elemente aufgeführt, die für mindestens eine App identifiziert wurden.

Onlinematerial 4: Behavior Change Techniques aufgeschlüsselt für die einzelnen Apps.

**Behavior Change Technique (BCT)**	Relax Noise 3 – Tinnitus	Dr Mollin Tinnitus	Shades of Noise	TinniTrain	Tinnitus Notched Tunes	Tinnitus Relief App. Klangtherapie	White Noise for Sleep. Generator for Tinnitus	Tonal Tinnitus Therapy	Tinnitracks Tinnitus Therapie	ReSound Tinnitus Relief	Tinnitus DE	Widex Zen. Tinnitus Management	Kalmeda Tinnitus-App	tinnitus help	Tinnitus HQ	Track Your Tinnitus	Beltone Tinnitus Calmer	Starkey Relax	Tinnitus Masker	TINNITUS, WHITE NOISE, SCHLAF	UNITI Tinnitus Studie
1.1 Goal setting (behavior)										✓			✓				✓				
1.2 Problem solving													✓								
1.4 Action planning													✓								
1.6 Discrepancy between current behavior and goal													✓								
1.9 Commitment													✓								
2.2 Feedback on behaviour			✓	✓	✓			✓	✓	✓	✓		✓				✓				
2.4 Self-monitoring of outcome(s) of behaviour			✓													✓					
2.7 Feedback on outcome(s) of behaviour													✓								
3.1 Social support (unspecified)									✓				✓								✓
4.1 Instruction on how to perform the behavior									✓	✓		✓	✓				✓				✓
4.3 Re-attribution													✓								
5.4 Monitoring of emotional consequences													✓								
5.6 Information about emotional consequences									✓	✓		✓					✓				✓
7.1 Prompts/cues								✓		✓			✓			✓	✓				✓

BCTs aufgeschlüsselt für *n* = 21 untersuchte Apps. Einsatz der Behavior Change Technique Taxonomy v1 nach Michie et al. 2013. Aus Gründen der Lesebarkeit sind nur BCTs aufgeführt, die für mindestens eine App identifiziert wurden.

Fortsetzung **Onlinematerial 4:** Behavior Change Techniques aufgeschlüsselt für die einzelnen Apps.

**Behavior Change Technique (BCT)**	Relax Noise 3 – Tinnitus	Dr Mollin Tinnitus	Shades of Noise	TinniTrain	Tinnitus Notched Tunes	Tinnitus Relief App. Klangtherapie	White Noise for Sleep. Generator for Tinnitus	Tonal Tinnitus Therapy	Tinnitracks Tinnitus Therapie	ReSound Tinnitus Relief	Tinnitus DE	Widex Zen. Tinnitus Management	Kalmeda Tinnitus-App	tinnitus help	Tinnitus HQ	Track Your Tinnitus	Beltone Tinnitus Calmer	Starkey Relax	Tinnitus Masker	TINNITUS, WHITE NOISE, SCHLAF	UNITI Tinnitus Studie
7.7 Exposure													✓								
7.8 Associative learning													✓								
8.1 Behavioral practice/ rehearsal				✓					✓	✓		✓	✓				✓				
10.3 Non-specific reward									✓												
10.6 Non-specific incentive									✓												
10.9 Self-reward									✓												
11.2 Reduce negative emotions									✓												
13.2 Framing/reframing									✓	✓			✓				✓				
13.4 Valued self-identity													✓								
15.3 Focus on past success													✓								

BCTs aufgeschlüsselt für *n* = 21 untersuchte Apps. Einsatz der Behavior Change Technique Taxonomy v1 nach Michie et al. 2013. Aus Gründen der Lesebarkeit sind nur BCTs aufgeführt, die für mindestens eine App identifiziert wurden.
